# Progeria Presenting with Pyogenic Granuloma in Conjunctiva: A Case Report

**DOI:** 10.31729/jnma.8218

**Published:** 2023-07-30

**Authors:** Sanket Parajuli, Sadhana Sharma, Tina Shrestha

**Affiliations:** 1 Department of Ophthalmology, Reiyukai Eiko Masunaga Eye Hospital, Banepa, Kavrepalanchowk, Nepal; 2 Department of Vitreo-retina, B.P. Koirala Lions Center for Ophthalmic Studies, Institute of Medicine, Maharajgunj, Kathmandu, Nepal; 3 Department of Ophthalmology, Dhulikhel Hospital, Dhulikhel, Kavrepalanchowk, Nepal

**Keywords:** *case reports*, *genetic disorders*, *hutchinson-gilford progeria syndrome*, *pyogenic granuloma*

## Abstract

Hutchinson-Gilford progeria syndrome frequently exhibits stunted growth and premature ageing. Notable ocular characteristics can encompass a large number of ocular abnormalities. Here, we report a case of a 17-year-old female who presented with complaints of a red bump not resolving with medications in her right eye for 3 months. On excisional biopsy, the mass was found to be a pyogenic granuloma. The occurrence of pyogenic granuloma in a patient with progeria is extremely rare. This highlights the importance of considering atypical presentations and potential complications in patients with Hutchinson-Gilford progeria syndrome. This information can assist healthcare professionals in the early recognition and appropriate management of ocular conditions in individuals with Hutchinson-Gilford progeria syndrome.

## INTRODUCTION

Hutchinson-Gilford progeria syndrome (HGPS) is a rare hereditary disorder with a prevalence of one in 4-8 million new births.^1,2^ A common pathology in HGPS is the rise in hyaluronic acid excretion.^3^ Patients with HGPS often present with growth retardation, premature ageing, and accelerated degenerative changes in the integumentary, cardiovascular, and musculoskeletal systems.^4,5^ Ocular features can include prominent eyes, loss of eyelashes and eyebrows, ectropion, ptosis, dry-eye syndrome, corneal clouding, cataract, strabismus, retinal vascular changes, pigmentary retinopathy, and optic atrophy.^6^ This case report is unique as it describes the occurrence of pyogenic granuloma, a benign vascular skin lesion, in a patient with progeria. The combination of these two conditions is extremely rare and highlights the importance of understanding the specific challenges and considerations in diagnosing and managing HGPS.

## CASE REPORT

A 17-year-old female from Panauti, presented with complaints of a red bump not resolving with medications in her right eye for 3 months. On examination, her best-corrected visual acuity (BCVA) in both eyes was 6/6 with Snellen's visual acuity chart. External ocular examination showed excessive forehead wrinkling for age, thickened eyelids, pseudo-proptosis, superior sulcus deformity, lagophthalmos, lid lag in downgaze, and loss of eyelashes and eyebrows in both eyes.

**Figure 1:**
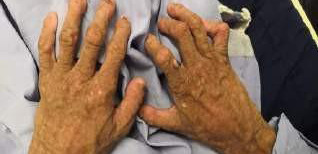
Wrinkled sclerodermatous skin.

Systemic examination revealed a typical senile look with craniofacial disproportion, frontal and parietal bossing called pseudo hydrocephaly, beaked nose, mandibular and maxillary hypoplasia, prominent scalp veins, protruding ears, thin lips, thin limbs, prominent and stiff joints, thin and high-pitch voice, generalized alopecia, absence of subcutaneous fat, thin and wrinkled 'sclerodermatous' skin, flecks like hyperpigmentation in sun-exposed areas, prominent superficial veins, and nail dystrophy (Figure 1).

**Figure 2:**
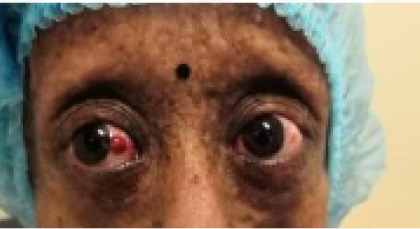
Pyogenic granuloma on the nasal aspect of the right eye.

Slit-lamp examination revealed a dry and keratinized ocular surface and blocked meibomian gland orifices in both eyes. Cornea was clear and lustrous. There was a small congested mass (3x4 mm in size), not moving with the conjunctiva in the bulbar conjunctiva of the nasal aspect of the right eye (Figure 2).

**Figure 3:**
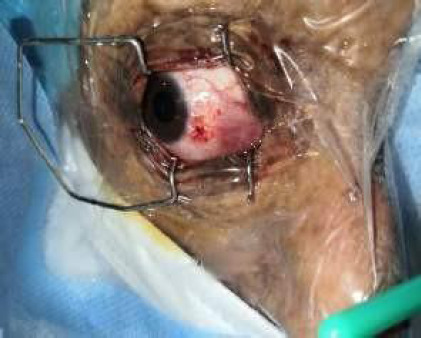
Excision of the lesion under topical anaesthesia.

There was grade 1 nuclear sclerosis in both eyes. Intraocular pressure was 15 mmHg and 13 mmHg in the right eye and left eye respectively, measured by air-puff tonometry. Fundus examination under mydriasis was normal in both eyes. Antenatal history was obtained which was uneventful, and delivery was normal at 40 weeks of gestation. Her parents gave no history of consanguinity of marriage. A general physical examination of the patient showed a height of 137 cm and a weight of 34 kg. Her pulse was 72/min, and her blood pressure was 110/70 mmHg. Because of the characteristic clinical presentation, the child was diagnosed with HGPS. An excisional biopsy of the mass was done, the histopathological report showed it to be a pyogenic granuloma (Figure 3).

Steroids and tear substitutes were given postoperatively for 3 weeks after excision. On follow-up, the lesion had healed properly with minimal conjunctival scarring and is under regular follow-up in our hospital.

## DISCUSSION

Currently, more than 142 patients with classical progeria syndrome i.e. Hutchinson-Gilford progeria syndrome have been reported worldwide.^3^ Progeria can present with a wide range of systemic and ocular manifestations. Our case presented with pyogenic granuloma, a benign vascular proliferation of immature capillaries that usually denotes sequelae of incomplete surgical or traumatic wound healing.^7,8^ In a review of ocular pyogenic granulomas, 87 out of 100 cases had predisposing factors, while 13 cases had unknown etiologies.^7^ A case series showed no specific predisposing risk for pyogenic granuloma.^9^ Similarly, in our case, the patient did not have any definite clinical history which would indicate the lesion as pyogenic granuloma. These findings suggest that, even though specific etiologies do not exist, the diagnosis of pyogenic granuloma should be considered with its typical clinical presentation of rapid growth and distinctive pink fleshy appearance. In addition to pyogenic granuloma, our case is diagnosed with HGPS, which in itself can present with a multiple arrays of ocular abnormalities.^10^ Many of the ocular pathologies are based on individual case reports and are absent in our case. Common findings are loss of eyebrows and eyelashes, cataracts, and lipodystrophy of the orbital fat resulting in superior sulcus deformity.

In summary, we have reported a patient with HGPS who demonstrated not only various orbital and ophthalmic manifestations of progeria but also a pyogenic granuloma on the conjunctiva that required an excision. The limitation of this case report is that genetic testing was not performed as it is not readily available and also the diagnosis can be clinical in HGPS. This case report contributes to the medical literature by providing insights into the ocular manifestations and diagnostic challenges associated with Hutchinson-Gilford progeria syndrome. It adds to the existing knowledge base, facilitates early recognition and appropriate management of ocular conditions, and improves the overall understanding of this rare genetic disorder among healthcare professionals.
